# The metabolic effect of fructose on normal rats in a mild dose with glucose and saccharose as control

**DOI:** 10.29219/fnr.v65.5589

**Published:** 2021-05-18

**Authors:** Ge Song, Wentao Qi, Yong Wang, Shaojie Pang, Yong Li

**Affiliations:** 1 Institute of Grain Quality and Nutrition Research, Academy of National Food and Strategic Reserves Administration, Beijing, People’s Republic of China; 2 Department of Nutrition and Food Hygiene, School of Public Health, Peking University, Beijing, People’s Republic of China

**Keywords:** fructose, glucose, saccharose, mild dose, metabolic effect, intestinal micro biota

## Abstract

**Aims:**

To study the metabolic effects of fructose, glucose and saccharose in a moderate dose by analyzing changes of blood indicators, pancreas inflammation, liver fat accumulation and intestinal microbiota in normal Sprague-Dawley (SD) rats.

**Subjects and methods:**

Six-week-old rats were assigned to four groups (*n* = 10), which were gavaged with normalsaline (Con), glucose dissolved in normal saline (Glu), saccharose-glucose dissolved in normal saline (Sac), and fructose dissolved in normal saline (Fru) for 20 weeks.

**Results:**

No significant differences in body weight and blood parameters including total cholesterol (TC), total triglyceride (TG), low-density lipoprotein cholesterol (LDL-C), high-density lipoprotein cholesterol (HDL-C), lipase (LPS) and free fatty acid (FFA) among the Con, Glu, Sac and the Fru group. The fructose can significantly (*P* < 0.05) decrease fasting and postprandial blood glucose increase compared to glucose, and the risk of pancreas inflammation and liver fat accumulation induced by fructose is lower than glucose in rats. We found there were no significant differences in intestinal microbial diversity. At the family level, rats in the Glu group had a relatively higher abundance of *Peptostreptococcaceae* and rats in the Fru group had a relatively higher abundance of *Bacteroidaceae*. Moreover, the proportions of *Peptostreptococcaceae romboutsia* and *Staphylococcus lentus* in the Glu group were significantly higher than in the Fru group, while the proportions of *Lachnospira*; *Lachnospiraceae blautia*, *Bacteroides* and *Cellulosilyticus* in the Fru group were significantly higher than in the Glu group. The concentration of isobutyric acid was relatively lower in all the sugar treated groups than in the Con. A significant decrease in isobutyric acid was found on comparing the Fru group to the Con group (*P* < 0.05).

**Conclusion:**

Fructose, glucose and sucrose made no significant changes on rats in body weight, blood indicators, organ index and bacterial diversity. Moreover, fructose can potentially attenuate fasting and postprandial blood-glucose increase, pancreas inflammation and liver-fat accumulation when compared to glucose in mild doses. The relative abundance of six kinds of bacterial genera was found significantly different between rats fed on fructose and glucose.

## Popular scientific summary

- The effects of fructose on health have been controversial.
- In this research we provide a better understanding on the metabolic effects of consuming reasonable quantities of fructose, glucose and saccharose.
- We found no significant differences in body weight and blood parameters among these three kinds of sugars.
- The fructose significantly decreases fasting and postprandial blood glucose increase compared to glucose, and the risk of pancreas inflammation and liver fat accumulation induced by fructose is lower than glucose in rats.
- At the family level, Rats in the fructose group had a relatively higher abundance of *Bacteroidaceae*, moreover, the proportions of *Lachnospira*; *Lachnospiraceae blautia*, *Bacteroides* and *Cellulosilyticus* were significantly higher than glucose group.

## Introduction

Fructose is a monosaccharide with the same chemical formula as glucose while they differ in chemical structure. One molecule of fructose and one molecule of glucose compose saccharose through a 1-4 glycoside bond. Fructose is present in many fruits and vegetables and exists in food (1). Fructose consumption is popular in western diets because of the introduction and generalised use of high-fructose corn syrup (HFCS) by the food industry (2, 3). However, it is not on the rise in other parts of the world.

Epidemiologic studies have shown a parallel increase in fructose consumption with metabolic syndrome traits (4). High-sugar consumption is well known to contribute to the worldwide epidemics of obesity, diabetes, and associated with cardiometabolic risks. As a result of its unique metabolic properties, the fructose component of sugar is widely considered to be harmful (5, 6). It has been reported currently that long-term consumption of high-level fructose elicits remarkable morphological and functional modifications of white adipose tissue in rats, which is closely related to obesity and insulin resistance (7, 8). Some results suggested that overconsumption of fructose upregulated pro-inflammatory markers and decreased antioxidative capacity in the visceral adipose tissue (VAT) of young female rats which consequently lead to the development of adiposity (9). Many studies have demonstrated that normal rats fed a fructose-enriched diet develop hypertension (10, 11). Studies have found fructose-fed rats showed blood pressure values similar to the control rats, but they had increased glycemia and insulinemia. Fructose-feeding negatively affects antioxidant capacity in the blood of hypertensive rats while it improves this capacity in the liver (10). A pilot study indicated a moderate reduction of dietary fructose and/or general sugar intake might positively affect BMI in overweight and obese children (12). Another report stated that the high-fructose diet consumption resulted in stronger bones with enhanced micro-architecture than the high-glucose diet (13). It has been noted that high-fructose corn syrup (HFCS) and saccharose increased postprandial triglycerides compared to fructose (14).

The intestinal microbial community has been linked with several metabolic diseases, including diabetes, nonalcoholic fatty liver disease and obesity (15, 16). It was reported that a high-glucose, or fructose diet causes changes in the gut microbiota and metabolic disorders in mice without body weight change (16). The intestinal metabolite profiles associated with fructose-feeding resulted in reduced bacterial diversity and seems to have more potential influences in host metabolic disturbances than glucose (17, 18). Results showed that high-fructose diets induced changes in the gut microbial community to enhance intestinal permeability and promote the leakage of lipopolysaccharides (LPS) into circulation by decreasing the expression of intestinal tight junction proteins (15, 19).

Therefore, it appears that the effects of fructose on health have been controversial. Additional research is needed to determine the adverse effects of consuming added sugars, including pure fructose (14). So far, most of the studies focused on the high-level consumption of fructose. There is limited research in comparing the effects of different sugar, including fructose, glucose and saccharose on induced changes to gut microbiota and the subsequent effects on metabolic diseases in low or middle levels. In this study, we fed normal healthy rats with a mild dosage of fructose over 20 weeks with glucose and saccharose as the control to evaluate the effects of pure fructose on blood indicators, pancreas inflammation, liver fat accumulation and intestinal flora, *etc.*

## Material and methods

### Materials

Standard substance of acetic acid, propionic acid, isobutyric acid and butyrate acid were purchased from Sigma-Aldrich (St. Louis, MO, USA). The antibodies perilinpin-1, occludin, β-actin and secondary antibodies were purchased from Abcam (Cambridge, MA, USA). Tight junction protein-1 (ZO-1) was obtained from MILLIPORE (Billerica, MA, USA). Tail-interacting protein (TIP-47) was purchased from Santa Cruz (Texas, CA, USA). Perilinpin-2 was obtained from Proteintech Group (Rosemont, IL, USA). ELISA Kit IL-10 and IL-6 were purchased from R&D System (Minneapolis, MN, USA).

### Animals

Six-week-old male Sprague-Dawley (SD) rats, purchased from Beijing Vital River Laboratory Animal Technology (Beijing, China), were housed in the laboratory of animal experiments at the Academy of National Food and Strategic Reserves Administration. Rats were caged and were maintained under controlled temperature (22 ± 2°C), humidity and airflow condition, with a 12-h on/off light cycle.

### Experimental design

After 1 week of adaptive breeding, rats were assigned to four groups (*n* = 10), which were gavaged with normal saline (Con), glucose dissolved in normal saline (Glu), saccharose-glucose dissolved in normal saline (Sac), and fructose dissolved in normal saline (Fru) for 20 weeks, with two rats being placed in each cage. Rats were kept under SPF conditions and provided with a standard maintenance diet (Beijing Keao Xieli Feed Co. Ltd., Beijing, China) and water *ad libitum*. The basal diet formula is displayed in Table 1. The nutrients in a normal diet are as follows:

**Table 1:** Formula of the experimental basal diet^a^ (g)

Ingredients	Content
Casein	187.25
L-methionine	3.02
Corn starch	449.72
Dextrin	200.37
Sucrose	69.20
Soybean oil	42.13
Mineral and vitamin^b^	48.30
t-BHQ^c^	0.01
Total	1,000

a Diet were produced according to AIN-93M diet.

b Mineral and vitamin mixture were prepare according to AIN-93M diet.

c t-BHQ, tert-butylhydroquinone.

#### Amino acids

Methionine + cystine (5.80 g/kg); lysine (8.90 g/kg); tryptophan (2.10 g/kg); arginine (9.90 g/kg); leucine (14.80 g/kg), isoleucine (7.40 g/kg), threonine (6.60 g/kg), valine (8.90 g/kg), histidine (4.90 g/kg), phenylamino acid + tyrosine (14.60 g/kg).

#### Vitamins

Vitamin A (7800.00 IU/kg), vitamin D (1200.00 IU/kg), vitamin E (67.00 mg/kg), vitamin K (5.00 mg/kg), vitamin B1 (10.00 mg/kg), vitamin B2 (15.00 mg/kg), vitamin B6 (10.00 mg/kg), vitamin B12 (0.02 mg), niacin (55.00 mg), pantothenic acid (22.00 mg), biotin (0.20 mg), choline (1,250 mg), folic acid (6.60 mg).

#### Minerals

Sodium (3.10 g/kg), magnesium (2.90 g/kg), potassium (7.40 g/kg), copper (11.40 mg/kg), iron (113.70 mg/kg), manganese (80.00 mg/kg), zinc (31.60 mg/kg), selenium (0.20 mg/kg), iodine (0.70 mg/kg).

#### Energy supply (%)

Protein 23.07%, fat 11.85%, carbohydrate 65.08% and total energy 3.40 kcal/g.

The WHO has recently proposed to halve the recommendation for free sugar intake from 10% to 5% of energy intake which is about 0.83 g/kg/day, to reduce the incidence of obesity and dental caries (20). Therefore, the rats were gavaged with a dose of 5.3 g/kg/day glucose, saccharose and fructose based on the suggested equivalent dose conversion, respectively (21). And the same volume of normal saline was gavaged to the rats in the control (Con) group. Blood glucose, food consumption, and weight gain were recorded twice per week until the end of the study. The hepatosomatic index (HSI = weight of liver/total rat weight) and colonic length index (CLI = length of colon/total rat weight) were determined at the end of the experiment.

The animal ethics committee approved all guidelines and experimental procedures in the entire animal trial of the Academy of National Food and Strategic Reserves Administration with utilization permission from Beijing Municipal Science & Technology Commission (No. SYXK [Jing] 2019-0015) and agreed with the guidelines of experimental animal management of People’s Republic of China (Documentation number 55, 2001, Ministry of Health of PR China).

## Glucose level change after sugar administration

Fasting and postprandial serum glucose concentrations were measured at the 10 and 18 weeks, respectively. The fasting time was 12 h. Blood glucose levels measured with a glucometer (Johnson & Johnson, New Jersey, USA) at 0, 30, 60, and 120 min after different sugar administration.

## Blood serum analysis

After 20 weeks, the rats were fasted for 12 h and euthanized using CO_2_. Blood was collected into microfuge tubes by cardiac puncture. The blood samples were centrifuged at 3,000 g for 10 min, the serum was collected, which we froze at -80°C until biochemical analysis. The concentration of total cholesterol (TC), total triglyceride (TG), low-density lipoprotein cholesterol (LDL-C), high-density lipoprotein cholesterol (HDL-C) in blood serum were analyzed by a biochemistry analyzer (Roche Modular, Roche, USA), uric acid (UC), insulin (INS), aspartate transaminase (AST), alanine amiotransferase (ALT), lipase (LPS), superoxide dismutase (SOD), malondialdehyde (MDA) and free fatty acid (FFA) in blood serum were determined by assay kit (Beijing Sino-UK Institute of Biological Technology).

## Histological analysis

Histological analyses were performed after hematoxylin and eosin (H&E) staining. The liver and pancreas were fixed in 10% formalin after rats were sacrificed. The fixed tissues were embedded in paraffin and sliced into 5-μm sections. Then, the tissue sections were stained with H&E. Digital images were obtained using a BA-9000 L microscope (Osaka, Japan). All experiments and scores were performed in a blinded manner by a pathologist in the Department of Pathology at Peking University (22, 23). The criteria of pathological evaluation are divided into 4 categories: 0 = one; 1 = slight abnormality; 2 = mild abnormality; 3 = moderate abnormality; severe abnormality. The standard parameters include: liver (hepatocyte edema, steatosis of hepatocytes, hepatocyte necrosis, number of necrotic foci/10 10 times visual field, inflammatory cell infiltration in portal area and liver congestion); pancreas (number of islets/10 10 times field of vision, degree of islets size difference, degree of capillary dilation in islet, infiltration of inflammatory cells in islets and degree of irregular shape of islets); Colon (degree of epithelial cell injury, degree of inflammatory cell infiltration and degree of goblet cell reduction).

## Western blotting

Liver tissue and colonic mucosal samples were used for protein analysis. Briefly, the mucosal samples were washed with serum-free media, followed by extraction of total using the cell lysis buffer (Cell Signaling Technology, Danvers, MA, USA). The lysates were centrifuged at 12,000 r/min for 20 min at 4°C. Protein concentration was determined using the Bradford assay (Bio-Rad, Hercules, CA, USA), and equal amounts of proteins were subjected to sodium dodecyl sulfate-polyacrylamide gel electrophoresis and transferred to nitrocellulose membranes (Bio-Rad). After blocking, specific antibodies against perilipin, ADRP, TIP-47, occluding, ZO-1, and β-actin were analyzed to probe the blots. Finally, each protein was detected and quantified using the ODYSSEY FC imaging system (Gene Company Limited, NE, USA). Relative optical density (arbitrary units) was obtained after normalizing to the control groups and β-actin band intensities in each series. Each test was performed in triplicate.

## Short-chain fatty acid analysis

Gas chromatography (Agilent 6890, CA, USA) was used to analyze SCFA in colonic contents by applying 2-ethylbutyric acid as the internal standard, acetic, propionic, butyric and isobutyric acids as standards. Frozen colonic contents (0.5 g) were diluted with ice-cold physiological saline (1 mL). All colonic contents were mixed in a vortex and centrifuged at 10,000 r/min for 5 min. The supernatant was collected and 25% (w/v) metaphosphoric acid solution (9:1) were added. Colonic contents were incubated overnight at 4 °C, centrifuged at 10,000 r/min for 5 mins afterwards and the supernatant was collected. A DB-FFAP chromatographic capillary column (30 m × 0.25 mm × 0.5 µm; Agilent) was applied to determine short chain fatty acids (SCFA) under the following procedure: initial temperature of 80 °C for 0.5 min, heating to 130 °C at 5 °C/min maintained for 2 min, and heating to 240 °C at 20 °C/min for 1 min. The signal was detected at 270 °C with a flame ionization detector (FID).

## ELISA analysis

Tumor necrosis factor- α (TNF-α), IL-6, MIP-2 and IL-10 levels in serum were measured using ELISA development kits (R&D System, Inc., Minneapolis, MN, USA) and a Multiskan FC microplate reader (Thermo Fisher, Shanghai, China) according to the manufacturer’s instructions.

## Gut microbiota analysis

Fresh colonic contents of rats were collected at week 20 and immediately stored at -80°C until processing. For microbial community analysis, DNA extraction and the V3–V4 hypervariable region of the 16S rRNA gene amplification were carried out using a MiSeq (Illumina, San Diego, CA, USA).

Paired-end reads were assembled by FLASH. Clustering of 16S rRNA operational taxonomic units (OTUs) was defined at ≥97% sequence homology using CD-HIT-OUT and identified using rDNA Tools, based on reference dataset from the Ribosomal Database Project. Finally, taxonomic composition was assigned using Quantitative Insights Into Microbial Ecology (QIIME). Weighted and unweighted Unifrac distance matrices were applied to measure diversities. Principal coordinate analysis plots and Unweighted Pair Group Method with Arithmetic mean cluster were visualized using KRONA software, and cladograms were produced by LEfSe software.

## Statistical analysis

The results were expressed as the means ± standard deviation (SD) of triplicate measurements using SPSS 18.0. The statistical significance of data was determined using one-way analysis of variance (ANOVA), followed by Dunnett’s contrast, and *P* < 0.05 was considered significant.

## Results

### Effect of added sugars on body weight, feed intake, organ and blood parameters

**Fig. 1:**
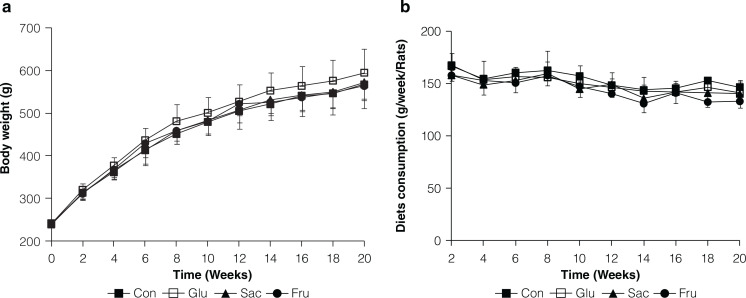
Body weight and diets consumptions of rats in Con, Glu, Sac and Fru groups. (a) Body weight changes during 20 weeks of feeding; (b) Diets consumption changes during 20 weeks of feeding. Data are expressed as the means ± SD, *n* = 10 per group.

Rats gavaged with fructose, saccharose, or glucose had overall similar weight gains to those gavaged with saline over the 20 weeks (Fig. 1a). The weight gain of rats in the Glu group had a higher trend than the other groups; however, there was no significant difference during the whole period (*P* > 0.05). The feed intake of rats in different groups also had no significant difference (*P* > 0.05) (Fig. 1b). The concentration of blood UA was found higher in the Glu group than the Sac or Fru group, while the difference was not significant (*P* > 0.05). And no significant (*P* > 0.05) difference was found in TC, TG, HDL-C, LDL-C, LPS, and FFA (Table 2).

**Table 2:** Blood serum parameters of rats in Con, Glu, Sac and Fru groups

Items	Con	Glu	Sac	Fru	F	P
TC	2.8±0.5	2.4±0.3	2.4±0.5	2.4±0.4	1.744	0.178
TG	1.3±0.3	1.8±0.6	1.6±0.8	1.7±0.7	0.668	0.578
HDL-C	1.7±0.2	1.5±0.2	1.6±0.2	1.6±0.2	0.599	0.621
LDL-C	0.7±0.1	0.5±0.1	0.5±0.1	0.6±0.2	1.533	0.226
LPS	67.3±10.9	64.9±9.9	62.0±11.2	63.3±6.9	0.410	0.747
FFA	0.4±0.1	0.5±0.1	0.5±0.1	0.5±0.1	0.841	0.482
UA	242.3±94.6	283.8±162.6	170.1±73.3	168.0±64.5	2.605	0.069

Treatments: CON = a basal diet gavaged with normal saline; Glu = a basal diet gavaged with glucose dissolved in normal saline; Sac = a basal diet gavaged with saccharose dissolved in normal saline; Fru = a basal diet gavaged with fructose dissolved in normal saline.

Each mean represents 5 replicate cages with 2 rats per cage (*n* = 10).

## Effect of added sugars on blood glucose and insulin level

**Fig. 2:**
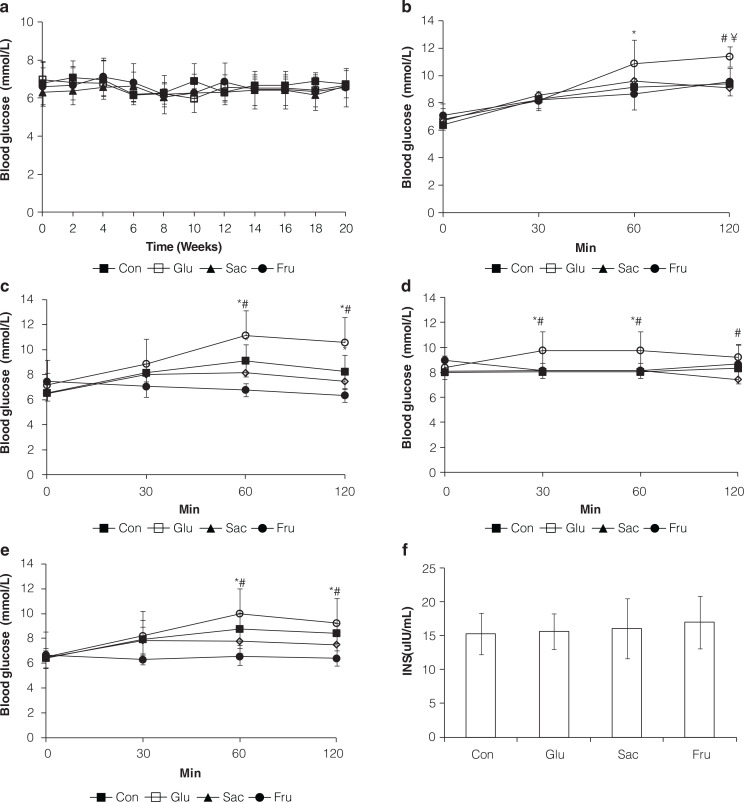
Glucose and insulin level alteration of rats in Con, Glu, Sac and Fru groups. (a) Glucose level changes along time during 20 weeks of feeding; (b) Postprandial glucose level changes without a 12 h fasting on week 18; (c) Glucose level changes with a 12 h fasting on week 10; (d) Postprandial glucose level changes without a 12 h fasting on week 10; (e) Glucose level changes with a 12 h fasting on week 18; (f) Insulin levels in the serum of rats on week 20. Data are expressed as the means ±SD, *n* = 10 per group; *P < 0.05 vs. control group; ^#^ *P* < 0.05 vs. fructose group; ^¥^ *P* < 0.05 vs. saccharose group.

The levels of fasting glucose of all the rats in different groups were determined every 2 weeks, and no significant differences were found from the beginning to the end (Fig. 2a). Fasting and postprandial serum glucose concentrations were measured at 10 and 18 weeks, respectively. The rats in the Glu group had significantly higher fasting and postprandial glucose levels than the rats of the Con and the Fru groups at 60 and 120 min (*P* < 0.05) (Fig. 2b–e). The glucose levels of the Sac group were found higher than Con group only under postprandial conditions (*P* < 0.05) (Fig. 2c, e). And there were almost no differences between the glucose levels of the Fru and the Con rats (Fig. 2b, d). Insulin concentration of rats in different groups was determined and showed no significant (*P* > 0.05) difference at the end of the experiment (Fig. 2f).

## Effect of added sugars on the pancreas of rats

**Fig. 3:**
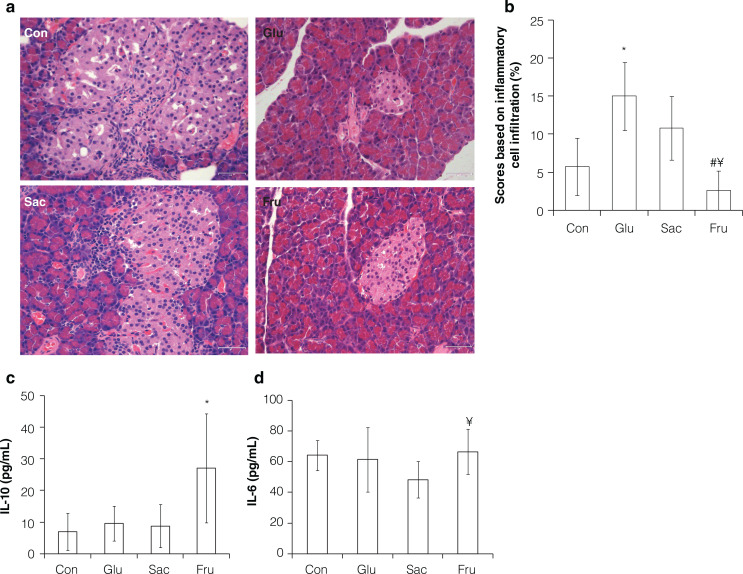
Effects of different sugar on pancreas of rats in Con, Glu, Sac and Fru groups. (a) Representative micrographs of HE-stained sections of rat pancreas (40×); (b) Scores based on inflammatory cells infiltration. Data represent means ± SD (*n* = 5); (c) IL-10 concentration in serum of rats. Data represent means ± SD (*n* = 8); (d) IL-6 concentration in serum of rats. Data represent means ± SD (*n* = 8). *P < 0.05 vs. control group; ^#^ *P* < 0.05 vs. glucose group; ^¥^ *P* < 0.05 vs. saccharose group.

The effects of different sugars on pancreatic pathology were analyzed and scored on the basis of available literature (22, 23). Tissue necrosis and inflammatory cell infiltration can be found in the pancreas of rats in the Glu and the Sac groups, while the pancreatic constructions of rats in the Con and the Fru groups apparently had no pathological damage (Fig. 3a). Scores based on the inflammatory cell infiltration of the Glu group were significantly higher than that of the Con group (*P* < 0.05). In contrast, the Fru group showed no significant difference compared with the Con group. Moreover, scores of the Fru group were significantly lower than those of both Glu and Sac groups (*P* < 0.05) (Fig. 3b).

To test whether the added sugar has the potential of inducing pancreatitis, the TNF-α, MIP-2, IL-10 and IL-6 levels in the serum of rats were further determined. With a significant increase, the IL-10 concentration of the Fru group were 3.87, 2.83, and 3.10 times of rats in the Con, the Glu and the Sac groups, respectively (*P* < 0.05) (Fig. 3c). Interleukin-6 (IL-6) concentration showed no significant difference between the Fru group and the Con group, while the Fru group rats showed a 27.4% increase of IL-6 level than those in the Sac group (Fig. 3d). No significant differences were found among all the groups where TNF-α and MIP-2 were concerned (Data were omitted).

## Effect of added sugars on the liver of rats

**Fig. 4:**
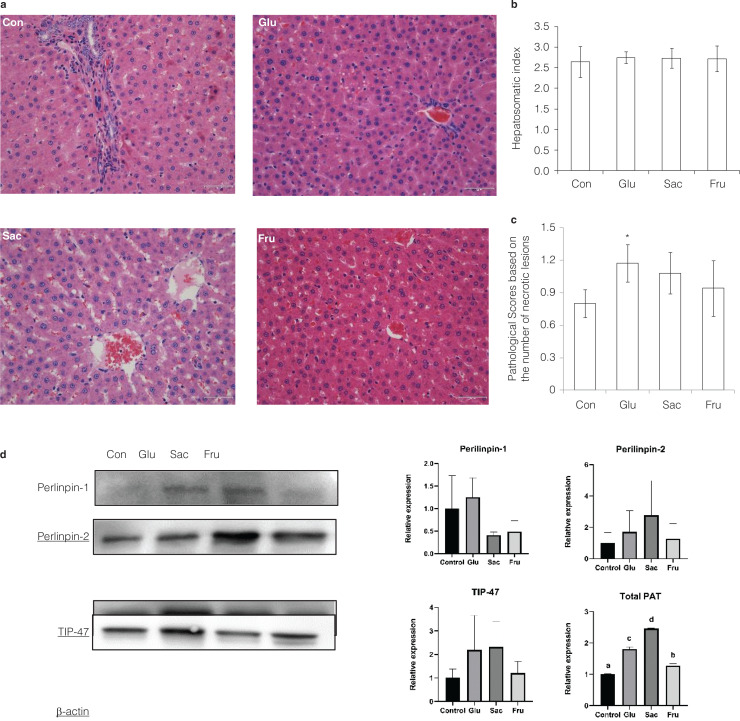
Effects of different sugar on livers of rats in Con, Glu, Sac and Fru groups. (a) Representative micrographs of HE-stained sections of rat liver. (40×); (b) Hepatosomatic index of rats at the end point of the experiments; (c) Pathological scores of liver based on the number of necrotic lesions. Data represent means ± SD (*n* = 5). *P < 0.05 vs. control group. (d) Western blot analysis of Perilipin-1, ADRP(Perilipin-2), and TIP-47expression in liver. Band densities were quantified by densitometry analysis. Data are presented after normalization to β-actin level. Data represent means ± SD (*n* = 3). Different superscripts indicated significant differences (*P* < 0.01, *n* = 3).

Glucose, sucrose and fructose had little effect on liver tissue based on the results of the hepatosomatic index (HSI) and parameters related to liver antioxidants including AST, ALT, SOD, and MDA (Fig. 4b and Table 3). All the same, a 10% microcellular steatosis was observed in the Glu and Sac groups, while no microcellular steatosis was observed in the Fru group. Some hepatocytes were necrotic in the Glu and the Fru groups, but no hepatocytes were necrotic in the Sac group. A small amount of inflammatory cell infiltration was observed in the portal area of the Glu group. No inflammatory cell infiltration was observed in the Sac and the Fru groups (Fig. 4a). The pathological score of the Glu group was significantly higher than the Con group (*P* < 0.05).

**Table 3:** Parameters related to liver antioxidant of rats in Con, Glu, Sac and Fru groups

Items	Con	Glu	Sac	Fru	F	*P*
AST	81.7±22.9	85.6±19.2	113.1±34.6	104.6±69.2	1.087	0.369
ALT	51.9±9.5	46.8±5.9	51.2±15.2	60.2±36.2	0.676	0.573
SOD	72.8±12.6	68.2±21.6	73.9±16.5	77.4±11.1	0.511	0.678
MDA	4.4±1.0	3.7±0.6	3.7±0.4	4.1±0.6	2.070	0.125

Treatments: CON = a basal diet gavaged with normal saline; Glu = a basal diet gavaged with glucose-dissolved normal saline; Sac = a basal diet gavaged with saccharose-dissolved normal saline; Fru = a basal diet gavaged with fructose-dissolved normal saline. Each mean represents 5 replicate cages with 2 rats per cage (*n* = 10).

In contrast, the differences in pathological scores between the Sac or Fru and the Con were not significant (*P* > 0.05) (Fig. 4c). The expression of the PAT family proteins, including Perilinpin-1, ADRP/Perilinpin-2 and TIP-47 were further examined. There were lower levels of all three proteins in the Con and the Fru groups as compared to the Glu and the Sac groups. And the results showed a significant decrease in total PAT protein expression including Perilinpin-1, ADRP and TIP-47 in the Fru group than both Glu and Sac groups (*P* < 0.01). The total PAT protein Con group was also significantly lower than in Glu and Sac groups (*P* < 0.01) (Fig. 4d).

## Effect of added sugars on colon pathological and tight junction protein expression results of rats

**Fig. 5:**
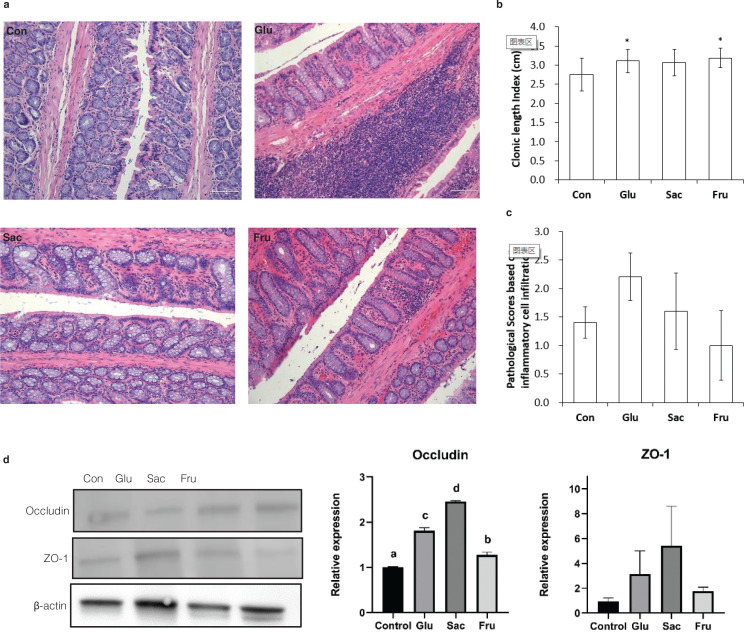
Effects of different sugar on colon of rats in Con, Glu, Sac and Fru groups. (a) Representative micrographs of HE-stained sections of rat colon. (40×); (b) Colonic length index of rats at the end point of the experiments, Data are expressed as the means ± SD, *n* = 10 per group; (c) Scores based on inflammatory cells infiltration. Data represent means ± SD (*n* = 5).*P < 0.05 vs. control group; (d) Western blot analysis of occludin and ZO-1 expression in colon. Band densities were quantified by densitometry analysis. Data are presented after normalization to β-actin level. Data represent means ± SD (*n* = 3). Different superscripts indicated significant differences (*P* < 0.01, *n* = 3).

At the end of the experiment, all the colonic length index (CLI) of rats in different sugar groups were found higher than those in the Con group, especially Glu and Fru groups (*P* < 0.05) (Fig. 5b). Inflammatory cell infiltration can be observed in the mucoderm and submucosa of the Glu and the Sac groups, while no obvious inflammatory cell infiltration was observed in the Con and the Fru groups (Fig. 5a). The pathological score of the Glu group was higher than the other groups, but the differences were not significant (*P* > 0.05) (Fig. 5c). The expression of cellular tight junction proteins Occludin and ZO-1 were further examined by the western blot. The Glu, Sac and Fru groups had higher Occludin expression in the colon than the Con rats (*P* < 0.05, Fig. 5d). However, there were no significant differences among all the groups in ZO-1 expression (*P* > 0.05, Fig. 5d).

## Effect of added sugars on gut microbiota

**Fig. 6:**
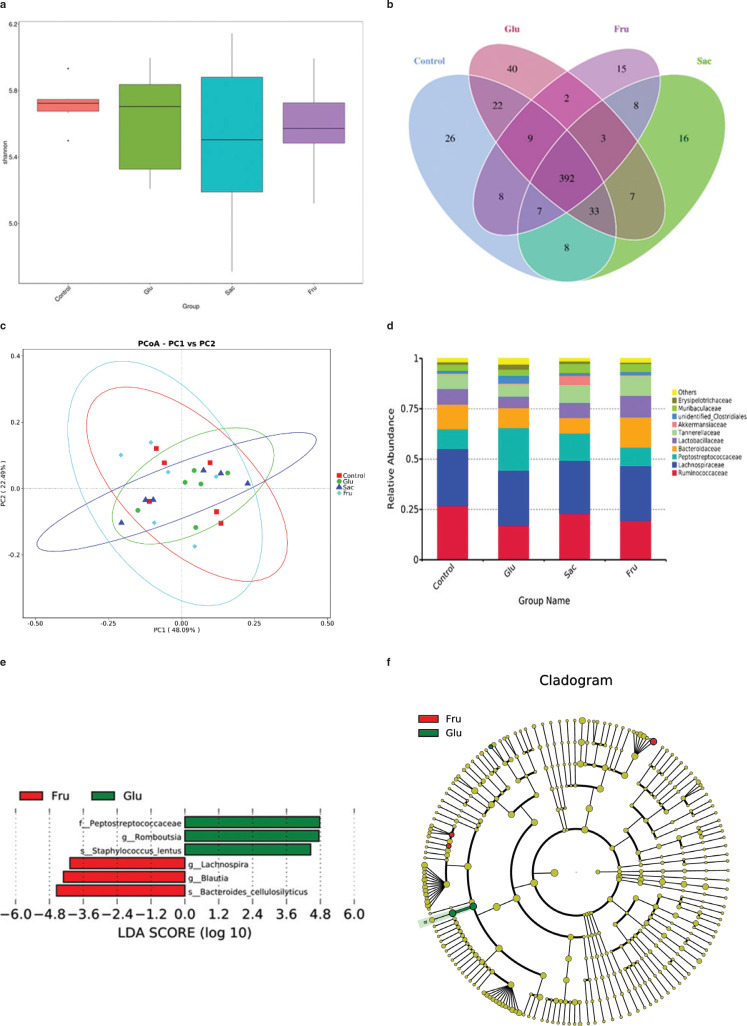
Analysis of the gut microbial community by 16S rRNA pyrosequencing from feces of Con, Glu, Sac and Fru groups. (a) Shannon’s diversity indices; (b) A Venn diagram displaying the degree of overlap of bacterial OTUs among the four groups; (c) principal coordinate analysis (PCoA) generated using a weighted Unifrac distance metrics. (d) Relative abundances plot of bacterial family; (e) Linear discriminant analysis (LDA), along with effect size measurements, was applied to identify enriched bacterial genera. Most enriched and depleted genera (LDA score (log10) > 2.0 were consider significant) in the Fru (red) vs.Glu (green); (f) Relative abundance cladogram of bacterial taxa. Data represent means ± SD (*n* = 6).

To compare community structure and similarity of gut microbiota among the four different groups, 16S RNA analysis was carried out in this research. Significant differences were not found in Shannon’s diversity indices among the four groups (Fig. 6a). The shared operational taxonomic units (OTUs) for different groups were determined via the Venn diagram (Fig. 6b). A total of 392 OTUs (65.8%) were detected in all four groups. The Glu group had a unique OTU (40) compared to the other groups, including the Con (26), Sac (16) and the Fru (15). The different OTUs between the Con and the Glu, the Con and the Sac, the Con and the Fru were 101, 99 and 112, respectively. The difference between Fru and Glu, Fru and Sac, Glu and Sac were 100, 98 and 112, respectively. Principal coordinate analysis (PCoA) on the taxonomic levels (Fig. 6c) indicated that with a moderate dose of added sugar, all the groups (Con, Glu, Sac and Fru) have similar micro biota profiles. At the family level, the Glu group had a relatively higher abundance of *Peptostreptococcaceae* and the Fru group had a relatively higher abundance of *Bacteroidaceae* (Fig. 6d). Interestingly, we observed higher proportions of *Peptostreptococcaceae romboutsia* and *Staphylococcus lentus* in the Glu group than in the Fru group (*P* < 0.05), while the proportions of *Lachnospira – Lachnospiraceas Blautia, Bacteroides* and *Cellulosilyticus* in the Fru group were significantly higher than in Glu group (*P* < 0.05) (Fig. 6e and f). Short-chain fatty acids (SCFA) in the colonic contents of rats in the Con, Glu, Sac and the Fru groups were further examined: no significant differences (*P* > 0.05) were found in acetic acid, propionic acid, butyric acid and total SCFA in all the groups (Table 4). However, the concentration of isobutyric acid was relatively lower in all the sugar treated groups than in the Con. There was a significant decrease in the Fru group compared to the Con group (*P* < 0.05).

**Table 4:** Short chain fatty acids concentrations in feces of rats in Con, Glu, Sac and Fru groups (µg/mg)

Items	Con	Glu	Sac	Fru
Acetic acid	1492.2±887.34	1317.6±677.11	1186.25±473.47	1060.67±296.54
Propionic acid	322.3±209.02	336.19±207.42	294.21±125.6	234.39±85.35
Isobutyric acid	489.72±570.66^b^	279.13±208.8^ab^	177.6±247.71^ab^	55.66±36.17^a^
Butyric acid	308.14±299.7	303.53±276.08	339.82±235.78	292.63±74.74

^a,b^Means within a row with no common superscript differ significantly (*P* < 0.05).

Identical small letter superscripts indicate no significant difference (*P* > 0.05). (*n* = 10).

## Discussion

The effects of sugar on human health has always been a hot topic. As a kind of popular additive sugar, fructose has been controversial from the beginning. A comparative research of the effects of fructose, glucose and saccharose on metabolic functions and gut microbiota was performed in this study. Different from many other studies (9–11), added sugar was fed to healthy rats by gavage every day for 20 weeks. And the dosage was determined on the basis of WHO recommendation and equivalent dose conversion. As a result, there were no significant differences in body weight and feed intake among the groups, although the body weight of rats in the Glu group was relatively higher. Some critical indicators in blood serum related to hypertriglyceridemia, hyperinsulinemia, fat accumulation such as TC, TG, HDL-C, LDL-C, LPS and FFA have not changed significantly in all the sugar treated groups. These findings indicate that moderate intake of fructose, as well as saccharose, has no influence on body weight gain and fat accumulation in rats.

Small and transient increases in plasma uric acid (UA) are likely found after consumption of sucrose-sweetened soft drinks in volumes as little as 355 mL (24). And gulping a fructose drink would be potentially more harmful than sipping it, because a unique characteristic of fructose metabolism is the synthesis of UA as a byproduct (4). In this study, we found that glucose intake can potentially increase the plasma UA with 16.9%, 66.5% and 68.5% in rats compared with control, saccharose and fructose. However, the increase was not significant (*P* = 0.069), which suggested that proper intake of fructose or saccharose would not stimulate the substantial rise in UA.

The effect on blood sugar is the most concerning part of sugar consumption. Data from animal experiments and human studies implicate added sugars, including saccharose and high-fructose corn syrup, lead to diabetes mellitus (25). But there is also evidence to the contrary that fructose-containing sugars independent of its form are associated with increased risk of type 2 diabetes (26). In this research, we found that both fasting and postprandial serum glucose concentrations in the Glu group were significantly higher than in the Con, Sac and Fru groups. The postprandial glucose level in the Sac group was also higher than that of the Con and the Fru groups. There were no significant differences found in comparing the Fru group to the Con group. Combined with a significantly higher level of anti-inflammation factor IL-10 in the Fru group, 5.3 g/kg/day based on WHO recommendation for 20 weeks, fructose consumption is more likely to inhibit inflammation as opposed to glucose or sucrose consumption in rats. No significant differences in insulin concentration in blood serum among all the groups were observed, which suggested no apparent damage to the islet by the three kinds of sugars in this dose.

Since the transport molecule (glut5) for fructose is absent in most cells, the liver and kidney are the main sites of fructose metabolism, which is possibly the reason that fructose stimulates the formation of lipids in the liver resulting in an increase in the circulation levels of TG (27) and causing high fructose-induced lipogenesis in rat liver (28). Our research however, found that fructose does not cause significant damage to the liver. There were no significant differences in the hepatosomatic index, pathological score and antioxidant ability among the Fru, Sac and the Con groups. The PAT proteins include perilipin-1, adipose differentiation-related protein (ADRP or perilipin-2) and TIP-47 (29), which are an ancient family of lipid droplet proteins regulating cellular lipid stores. This study found that the PAT family decreased significantly in the Fru group compared to both the Glu and the Sac groups. However, the PAT expressions of the Fru group were still significantly higher than those of the Con group. These results indicated that glucose and saccharose are more likely to contribute to the accumulation of fat in the liver than fructose in rats.

Short-chain fatty acids mainly refer to acetate, propionate and butyrate, which are metabolic end-products produced in the gastrointestinal tract by fermentation of nondegradable carbohydrates and proteins. It benefits in inhibiting intestinal inflammation (30, 31). Therefore, gut microbiota diversity and microbiota-derived SCFAs play essential roles in intestine health (32, 33). Mice on a high sugar diet lead to SCFA and microbial diversity reduction and thus increasing gut permeability and susceptibility to colitis (33, 34). In this research we found that in the rat intestinal tract, fructose leads to dramatic changes in the gut composition at the genus level as compared to glucose. However, there was no significant difference in microbial diversity and total SCFA contents. Tight junction proteins regulate gut permeability in the colonic cell, such as occludin and ZO-1 (15). Upregulated expression of occludin was found in this study. Interestingly, the colon length index of the Glu and Fru group (Fig. 5b) are higher than the Con and Sac group, which indicates the moderate dose of glucose and fructose may benefit colon health in SD rats. All these results mean that fructose, glucose and saccharose have no adverse effects on intestinal health under a mild dose.

## Conclusion

Many reports indicated that long-term consumption of fructose at a high level elicits adverse effects on body, such as obesity, diabetes and NAFLD. In this paper, we focused on the impact of fructose on normal-weight rats at a reasonable and moderate dose, with glucose and saccharose as control. Our results demonstrated that fructose, glucose and sucrose made no significant changes on rats; such as body weight, blood indicators, organ index and bacterial diversity. Moreover, fructose can potentially attenuate fasting and postprandial blood-glucose increase, pancreas inflammation and liver-fat accumulation when compared to glucose in mild doses. The relative abundance of six kinds of bacterial genera was found significantly different between rats fed on fructose and glucose. Based on these data, we have an understanding on the metabolic effects of consuming reasonable amounts of fructose compared with glucose and saccharose in normal subjects.

## Ethics approval and consent to participate

The animal experiments in this paper have received local approval of the Ethic Committee of Academy of National Food and Strategic Reserves Administration.
